# The American Medical Almanac for 1846; Containing Statistics of the Various Medical Colleges, Hospitals, Dispensaries, Etc. of the United States, Together with Other Information of Value to the Physician and Student

**Published:** 1847-11

**Authors:** 


Art. XI.—*The American Medical Almanac for* 1846; containing Statistics of the various Medical Colleges, Hospitals, Dispensaries, etc. of the United States, together with other information of value to the Physician and Student. Philadelphia: Lindsay and Blakiston.

The title expresses the character of this publication, the interest of which to all classes of medical men will be at once perceived. Every physician ought to be in possession of a copy of it, embracing as it does statistics to which all must frequently have occasion to refer. The admirable Code of Medical Ethics, adopted by the late Medical Convention, is contained in the present volume of the Almanac, and of itself is worth more than the cost of the work.

